# Supplementary material for: Trait-like visual cortical hyperactivity in trait anxiety
Source: Nat Commun. 2025 Dec 20;17:794. doi: 10.1038/s41467-025-67480-3 (PMC12824385; doi:10.1038/s41467-025-67480-3)
Supplement: Supplementary file 1 — Supplementary Information [file 41467_2025_67480_MOESM1_ESM.pdf]

Supporting Online Material for

Trait-like visual cortical hyperactivity in trait anxiety

Zhaohan Wu[1*], Yuqi You[1, 2*], Joshua A Brown[3], Raymond J. Dolan[4], & Wen Li[1, 3**]

[1] Department of Psychology, Florida State University, Tallahassee, FL, USA

[2] Department of Psychology and Behavioral Sciences, Zhejiang University, Hangzhou, China

[3] Louis A. Faillace, MD, Department of Psychiatry and Behavioral Sciences, University of Texas Health Science Center, Houston, USA

[4] Max Planck-University College London Centre for Computational Psychiatry and Ageing Research, University College London, London, UK

*Equal contributor

Corresponding author: Wen Li; **Email: wen.li.1@uth.tmc.edu

This PDF file includes:

Supplemental analyses

Figs. S1-S4

Tables S1-S2

Supplementary References

Experiment 1

Additional Analyses

Correlation between VEPs and trait anxiety for CS+ and CS- stimuli

Effects of conditioning on VEPs were reported in our earlier study (*1*). Here, to evaluate the effect of conditioning on the correlation between trait anxiety and P1/C1-N1, we also examined VEPs for CS+ and CS- stimuli separately at all 3 time points. As shown in **Table S1**, correlation was comparable for the two types of stimuli at all 3 time points, ruling out the effect on conditioning.

Table S1 correlation between VEPs and trait anxiety for CS+ and CS- stimuli

**Correlation coefficients (*p values*)**		**Time**		
		Time 1 (baseline)	Time 2 (Day 1 post-conditioning)	Time 3 (Day 16 Post-conditioning)
M-selective stimuli (P1)	CS+	-0.356 (*.014*)*	-0.382 (*.008*)*	-0.333 (*.047*)*
	CS-	-0.364 (*.012*)*	-0.409 (*.004*)*	-0.285 (*.092*)+
P-selective stimuli (C1-N1)	CS+	0.351 (*.016*)*	0.374 (*.010*)*	0.379 (*.023*)*
	CS-	0.370 (*.010*)*	0.384 (*.008*)*	0.358 (*.032*)*

**p<0.05 two tailed, +p<0.1 two tailed.*

Comparison of correlation between trait anxiety and ERP components at different times

The correlation coefficients between trait anxiety and P1/C1-N1 at 3 time points were compared using Hittner's method based on Monte Carlo simulation (*2*). The analysis revealed no significant differences in the correlation coefficients between trait anxiety and P1 components across the three time (*p*'s > .44). Similarly, another analysis indicated no significant differences in the correlation coefficients between trait anxiety and C1-N1 components across the three times (*p*'s > .60). Therefore, the strength of the correlations remained consistent over time.

Point-by-point analysis of correlation between VEPs and trait anxiety

To delineate the precise time course of the association between VEPs and trait anxiety, we correlated amplitude at each data point within the first 200 ms with trait anxiety. We applied a permutation package in R

that was designed to overcome Type I error from multiple comparisons (*3*) to determine the minimal window of consecutive data points with significant correlation ($p < 0.05$). Accordingly, at Time 1 (pre-conditioning baseline), significant time windows for M- and P-selective stimuli were identified as 74-200 ms, $p = 0.005$ and 86-175 ms, $p = 0.011$, respectively. At Time 2 (immediately post-conditioning), significant time windows for M- and P-selective stimuli were identified 42-200 ms, p = 0.002 and 86-171 ms, $p = 0.009$, respectively. At Time 3 (Day 16; 15 days post-conditioning), significant time windows for M- and P-selective stimuli were identified as 78-200 ms, $p = 0.02$ and 86-160 ms, $p = 0.029$, respectively.

<u>Correlation between P1 and C1-N1</u>

The intriguing opposite patterns of correlation between trait anxiety and the P1 versus C1-N1 prompted us to explore the direct association between P1 and C1-N1. As shown in **Fig. S1**, a significant negative correlation was observed between P1 and (inverted) C1-N1 amplitudes at every time point, $r = -0.62$, $p < 0.001$, $r = -0.73$, $p < 0.001$, $r = -0.63$, $p < 0.001$ at Time 1, Time 2, and Time 3, respectively.

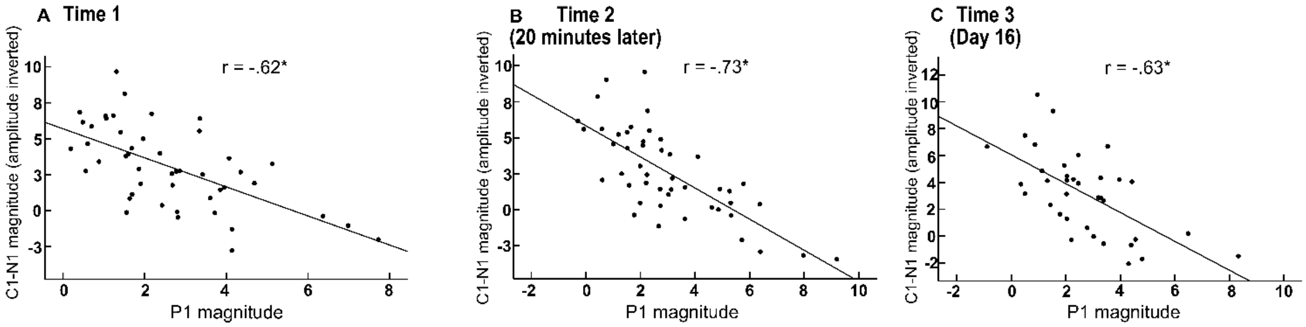

Figure S1 Correlation between P1 and C1-N1 at each time point (Experiment 1). A significant negative correlation was observed between P1 and (inverted) C1-N1 amplitudes at every time point: Time 1—Day 1 baseline **(A)**, Time 2—Day 1 post-conditioning **(B)**, and Time 3—Day 16 **(C)**; $r = -0.62$, $p < 0.001$, $r = -0.73$, $p < 0.001$, $r = -0.63$, $p < 0.001$ at Time 1, Time 2, and Time 3, respectively. Results were based on Pearson correlation analysis, and p values were two-tailed.

<u>Source-level analysis of the association between trait anxiety and VEPs</u>

Based on artifact-minimized VEP data from high-density EEG (hdEEG), we further conducted source-level analyses to confirm the involvement of early visual cortex (V1/V2) in the VEP-trait-anxiety association. We used exact low-resolution electromagnetic tomography (eLORETA; (4)), a linear inverse solution, to reconstruct cortical activity with scalp EEG data. The solution space consists of 6239 cortical gray matter voxels with a spatial resolution of 5 x 5 x 5 mm in a realistic head model (*5*) registered to standardized space from a digitized

MRI at the Montreal Neurologic Institute (MNI). We then estimated voxel-wise current density during the C1 and P1 windows, which was then regressed on the BIS scores. To minimize false-positive results in intracranial source localization, our laboratory has routinely applied two constraints in the analyses (*6-12*): (1) We constrained eLORETA analysis to the time windows and tests showing significant surface-level effects (*13*); and (2) we used Monte Carlo simulation based on the voxel spatial correlation inherent to the data to set a corrected statistical threshold of $p < .05$ FDR. Specifically, using Gaussian filter widths estimated from the current density, a voxel size of 5 × 5 × 5 mm^3, and a radius of 5 mm, we derived a corrected threshold of voxel-level $p < .005$ over four contiguous voxels for all three time points.

We observed significant associations between C1 magnitude and trait anxiety in early visual cortex at all three time points: T1—the V2: peak x = –40, y = –85, z = –10, $r = 0.44$, $k = 20$; FDR $p < .05$ (Gaussian filter widths: FWHMx = 1.74 mm, FWHMy = 1.66 mm, FWHMz = 1.72 mm; **Fig. S2** Top Left); T2—the V1: peak x = 20, y = –90, z = 5, $r = 0.43$, $k = 8$; FDR $p < .05$ (Gaussian filter widths: FWHMx = 1.98 mm, FWHMy = 1.68 mm, FWHMz = 1.74 mm; **Fig. S2** Top Middle); and T3—the V2: peak x = 10, y = –80, z = 45, $r = 0.43$, $k = 8$; FDR $p < .05$ (Gaussian filter widths: FWHMx = 1.75 mm, FWHMy = 1.74 mm, FWHMz = 1.65 mm; **Fig. S2** Top Right).

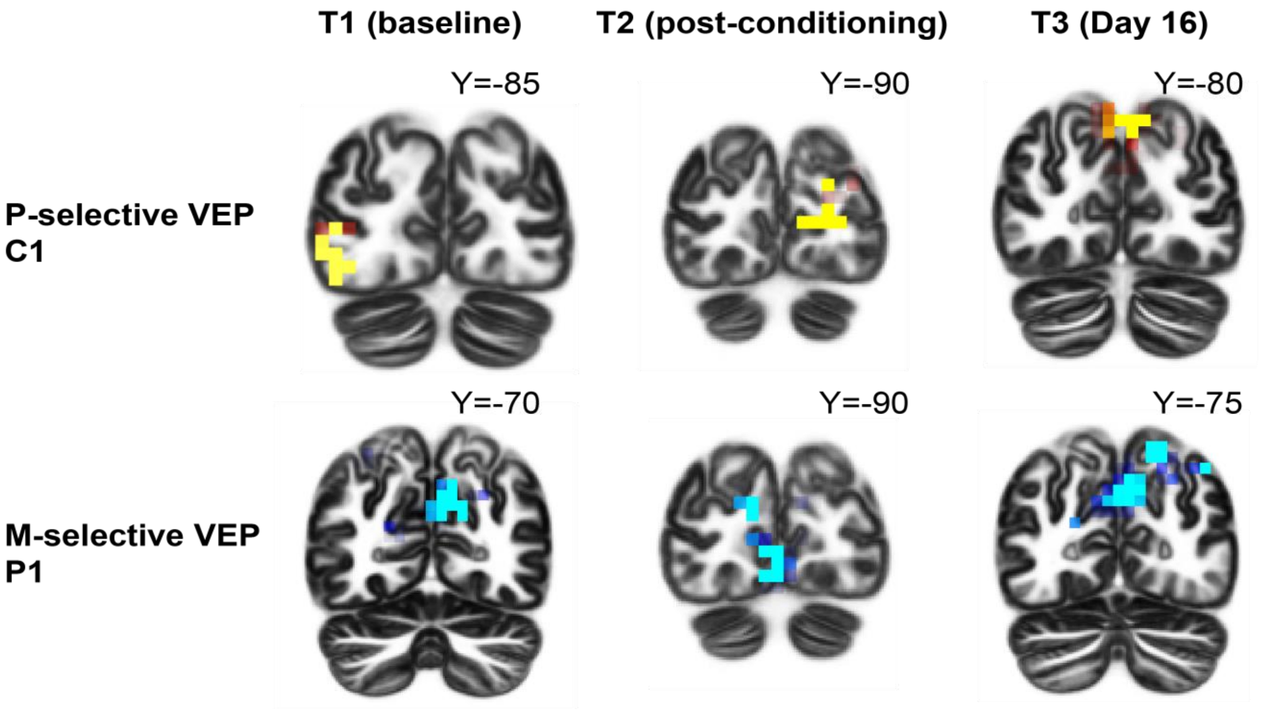

Figure S2 Early visual cortex localized for association of trait anxiety with P-selective C1 and M-selective P1 (Experiment 1; display threshold $p < .005$ uncorrected)

We also observed significant associations between P1 magnitude and trait anxiety in early visual cortex at all three time points: T1—the V2: peak x = 10, y = –70, z = 30, $r = -0.49$, $k = 22$; FDR $p < .05$ (Gaussian filter widths: FWHMx = 2.15 mm, FWHMy = 2.12 mm, FWHMz = 2.00 mm; **Fig. S2** Bottom Left); T2—the V1: peak x = –5, y = –95, z = 0, $r = -0.43$, $k = 13$; FDR $p < .05$ (Gaussian filter widths: FWHMx=1.57 mm, FWHMy = 1.56 mm, FWHMz = 1.48 mm; **Fig. S2** Bottom Middle); and T3—the V2: peak x = 10, y = –80, z = 40, $r = -0.43$, $k = 11$; FDR $p < .05$ (Gaussian filter widths: FWHMx = 1.96 mm, FWHMy = 1.89 mm, FWHMz = 1.82 mm; **Fig. S2** Bottom Right).

Figure S2 Early visual cortex localized for association between trait anxiety and VEPs (Experiment 1; display threshold $p < .005$ uncorrected)

Experiments 2 & 3

Additional Analysis

<u>Point-by-point analysis of correlation between VEPs and trait anxiety</u>

Similar to **Experiment 1,** we conducted point-by-point correlational analyses between VEP potentials and trait anxiety in the first 200 ms, delineating the precise time course of this association. We applied a permutation package in R that was designed to overcome Type I error from multiple comparisons (*3*). In **Experiment 2**, a significant time window was detected for the P-selective stimuli (58-105 ms, p = 0.038), but not for the M-selective stimuli (**Figure 2B**; grey box at the bottom). Similarly, in **Experiment 3**, a significant time window was detected for the P-selective stimuli (46-94 ms, $p = 0.023$), but not for the M-selective stimuli (**Figure 2D**; grey box at the bottom).

<u>Comparison of correlation between trait anxiety and VEPs</u>

We further compared the correlations between trait anxiety and P1/C1 in **Experiments 2** and **3**. We observed no difference in correlation coefficients between the two experiments, for either the correlation of trait anxiety with P1 amplitude ($p = 0.82$) or with C1 amplitude/magnitude ($p = 0.64$).

<u>Source-level analysis of the association between trait anxiety and C1</u>

Similar to **Experiment 1**, we submitted the P-selective C1 magnitudes evoked in **Experiments 2** and **3** to eLORETA and performed voxelwise correlation analyses with trait anxiety (BIS scores). For **Experiment 2**, significant associations between C1 magnitude and trait anxiety were observed in early visual cortex including V1 and V2: peak x = -20, y = –80, z = –20, $r = 0.45$, $k = 122$; FDR $p < .05$ (Gaussian filter widths: FWHMx = 2.57 mm, FWHMy = 2.57 mm, FWHMz = 2.38 mm; **Fig. S3** Left). For **Experiment 3**, significant associations between C1 magnitude and trait anxiety were observed in V2: peak x = –20, y = –55, z = 0, $r = 0.39$, $k = 10$; FDR $p < .05$ (Gaussian filter widths: FWHMx = 1.89 mm, FWHMy = 1.90 mm, FWHMz = 1.96 mm; **Fig. S3** Right).

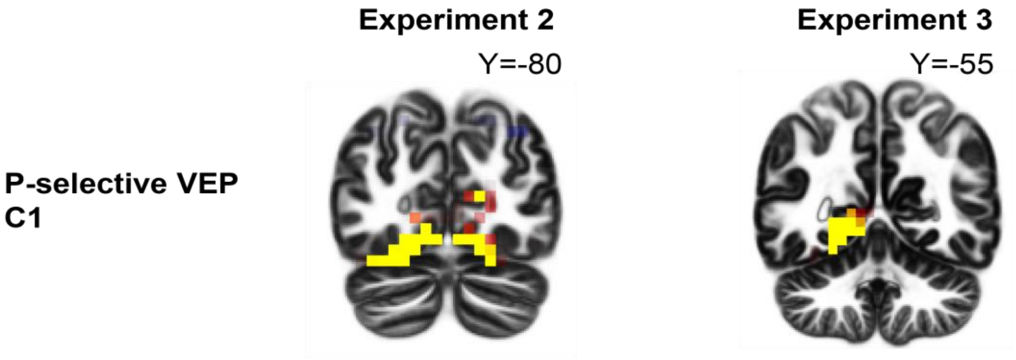

Figure S3 Early visual cortex localized for association between trait anxiety and P-selective C1 (Experiments 2 & 3; display threshold *p* < .005 uncorrected)

Bayes Factors for non-significant correlations

To further evaluate the non-significant correlation between trait anxiety and M-selective P1 amplitude, we performed Bayesian analyses to quantify the relative evidence for the null hypotheses. The resulting Bayes factors for **Experiments 2 & 3** were BF_{10} = 0.177 and 0.181, indicating the data were approximately 5.6 and 5.5 times more likely under the null hypothesis than under the alternative. This provides moderate evidence in favor of the absence of a relationship.

Experiment 4

Additional Analysis

Point-by-point analysis of correlation between VEPs and trait anxiety

Similar to the other experiments, we conducted point-by-point correlational analyses between VEP potentials and trait anxiety, using a permutation package in R to overcome Type I error from multiple comparisons (*3*). A significant time window was detected for the P-selective stimuli (101-140ms, *p* = 0.047), but not for the M-selective stimuli (**Figure 3B**; grey box at the bottom).

Effects of emotion on correlation between VEPs and trait anxiety

To explore whether the correlation between VEPs and trait anxiety was affected by emotion of the images, we conducted an analysis of covariance (ANCOVA) of emotion, SF, and trait anxiety on the VEPs. There was no interaction of emotion with SF and trait anxiety (*p* = 0.22). Specifically, the correlation between P1 and trait anxiety was comparable for fear, disgust, and neutral images in HSF: *r* = 0.37, 0.37, 0.36, respectively (*p*'s <

0.02), and similarly negligible for fear, disgust, and neutral images in LSF: *r* = 0.21, 0.16, 0.08, respectively (*p*'s > 0.20).

<u>Source-level analysis of the association between trait anxiety and P1</u>

Similar to previous experiments, we submitted the P-selective P1 to eLORETA and performed voxelwise correlation analyses with trait anxiety (BIS scores). A significant association between P-selective P1 magnitude and trait anxiety was observed in V1 and V2: peak x = 20, y = –85, z = 0, *r* = 0.47, *k* = 34; FDR *p* < .05 (Gaussian filter widths: FWHMx = 1.83 mm, FWHMy = 1.81 mm, FWHMz = 1.86 mm; **Fig. S4**).

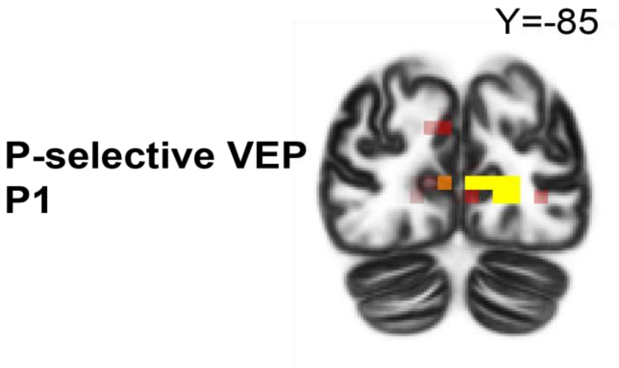

Figure S4 Early visual cortex localized for association between trait anxiety and P-selective P1 (Experiment 4; display threshold *p* < .005 uncorrected)

<u>Bayes Factors for non-significant correlations</u>

For the non-significant correlation between trait anxiety and M-selective P1 amplitude in **Experiment 4**, we performed Bayesian analyses to quantify the relative evidence for the null hypotheses. The resulting Bayes factor was BF_{10} = 0.299, indicating that the data were approximately 3.3 times more likely under the null hypothesis than under the alternative. This provides moderate evidence in favor of the absence of a relationship.

Correction for multiple comparisons across four experiments

The six correlational tests between trait anxiety and P-selective VEPs, across **Experiments 1-4**, each individually survived the threshold of *p* < .05 two-tailed. To enhance rigor, we further submitted the six *p*-values to Benjamin-Hochberg correction for multiple comparisons, which indicated that all six remained significant at the adjusted thresholds (**Table S2**).

Table S2 Exact p and q*values for correlational tests between trait anxiety and P-selective VEPs

i	*p*	*q**	*p < q**
1	0.0060	0.0083	TRUE
2	0.0090	0.0167	TRUE
3	0.0110	0.0250	TRUE
4	0.0170	0.0333	TRUE
5	0.0180	0.0417	TRUE
6	0.0260	0.0500	TRUE

Sex effect

To explore potential sex effects on the relationship between visual cortical hyperactivity and trait anxiety, data from all three samples were pooled and submitted to hierarchical liner regression analyses with sample (dummy-coded), sex, and BIS scores as regressors in the first level and sex-by-BIS interaction in the second level. Sample 1 was drawn from **Experiment 1** (with repeatedly measured VEPs from Time 1 and Time 2 averaged), Sample 2 was drawn from **Experiments 2** and **3** (with VEPs averaged between the two experiments), and Sample 3 was drawn from **Experiment 4**.

BIS significantly predicted P-selective VEPs (β = .34, $t(134) = 4.26$, $p < .001$, sr = .35), but not M-selective VEPs (β = –.01, $t(134) = –1.71$, p = .864, sr = –.02), after controlling for sex and sample. These results confirmed the main analyses. Importantly, the sex-by-BSI interaction was not significant for either P-selective VEPs (β = –.11, $t(134)$ = –0.30, p = .762, sr = –.03) or M-selective VEPs (β = –.02, $t(134)$ = –0.05, p = .960, sr = –.004), indicating no evidence that sex influenced the BIS-VEP relationship.

We further explored the BIS-VEP relation in male and female groups separately, with BIS and sample as regressors. In both groups, BIS significantly predicted P-selective VEPs (females: β = .35, $t(81)$ = 3.43, p = .001, sr = .36; males: β = .32, $t(51)$ = 2.65, p = .011, sr = .35), but not M-selective VEPs (females: β = –.02, $t(81)$ = –0.31, p = .760, sr = –.03; males: β = –.007, $t(51)$ = –0.07, p = .948, sr = –.01). These results suggest that the positive association between BIS and P-selective VEPs is consistent across sexes and that sex does not moderate the relationship between trait anxiety and VEPs.

Additional discussion

It is well-established that the C1 component originates from the earliest regions of the visual cortical hierarchy, particularly V1 (*14, 15*) and likely also V2 (*16, 17*). In keeping with that, our source-level analyses across all four

experiments consistently revealed significant associations between trait anxiety and P-selective VEPs in early visual cortex (V1/V2). While it is known that secondary visual cortex, such as the lingual gyrus in V2, can support the processing of emotional stimuli *(9, 12, 18),* recent findings, including our own, have also implicated V1 in threat processing (*10, 19, 20*). Representing a significant advance beyond prior findings, the current study indicates that early visual cortex (V1 and V2) not only flexibly encodes emotional value in sensory input but also exhibits a dispositional, general hyperfunctioning associated with trait anxiety.

Supplementary References

1. Y. You, J. Brown, W. Li, Human sensory cortex contributes to the long-term storage of aversive conditioning. *Journal of Neuroscience* **41**, 3222-3233 (2021).
2. J. B. Hittner, K. May, N. C. Silver, A Monte Carlo evaluation of tests for comparing dependent correlations. *The Journal of general psychology* **130**, 149-168 (2003).
3. J. Frossard, O. Renaud, Permutation tests for regression, ANOVA, and comparison of signals: the permuco package. *Journal of Statistical Software* **99**, 1-32 (2021).
4. R. D. Pascual-Marqui *et al.*, Assessing interactions in the brain with exact low-resolution electromagnetic tomography. *Philos Trans A Math Phys Eng Sci* **369**, 3768-3784 (2011).
5. M. Fuchs, J. Kastner, M. Wagner, S. Hawes, J. S. Ebersole, A standardized boundary element method volume conductor model. *Clinical neurophysiology* **113**, 702-712 (2002).
6. K. J. Clancy *et al.*, Posttraumatic Stress Disorder Is Associated with α Dysrhythmia across the Visual Cortex and the Default Mode Network. *eneuro* **7**, ENEURO.0053-0020.2020 (2020).
7. K. J. Clancy *et al.*, Transcranial stimulation of alpha oscillations up-regulates the default mode network. *Proceedings of the National Academy of Sciences* **119**, e2110868119 (2022).
8. E. C. Forscher, W. Li, Hemispheric Asymmetry and Visuo-Olfactory Integration in Perceiving Subthreshold (Micro) Fearful Expressions. *The Journal of Neuroscience* **32**, 2159-2165 (2012).
9. E. A. Krusemark, W. Li, Do all threats work the same way? Divergent effects of fear and disgust on sensory perception and attention. *Journal of Neuroscience* **31**, 3429-3434 (2011).
10. Y. You, J. Brown, W. Li, Human Sensory Cortex Contributes to the Long-Term Storage of Aversive Conditioning. *J Neurosci* **41**, 3222-3233 (2021).
11. Y. You, W. Li, Parallel processing of general and specific threat during early stages of perception. *Soc Cogn Affect Neurosci* **11**, 395-404 (2016).
12. E. A. Krusemark, W. Li, From early sensory specialization to later perceptual generalization: dynamic temporal progression in perceiving individual threats. *Journal of Neuroscience* **33**, 587-594 (2013).
13. R. Thatcher, D. North, C. Biver, Evaluation and validity of a LORETA normative EEG database. *Clinical EEG and Neuroscience* **36**, 116-122 (2005).
14. V. P. Clark, S. A. Hillyard, Spatial selective attention affects early extrastriate but not striate components of the visual evoked potential. *Journal of cognitive neuroscience* **8**, 387-402 (1996).
15. A. Martinez *et al.*, Involvement of striate and extrastriate visual cortical areas in spatial attention. *Nature neuroscience* **2**, 364-369 (1999).
16. J. J. Foxe, G. V. Simpson, Flow of activation from V1 to frontal cortex in humans: A framework for defining" early" visual processing. *Experimental brain research* **142**, 139-150 (2002).
17. P. Ossenblok, H. Spekreijse, The extrastriate generators of the EP to checkerboard onset: A source localization approach. *Electroencephalography & Clinical Neurophysiology: Evoked Potentials* **80**, 181-193 (1991).
18. W. Li, R. E. Zinbarg, S. G. Boehm, K. A. Paller, Neural and behavioral evidence for affective priming from unconsciously perceived emotional facial expressions and the influence of trait anxiety. *Journal of cognitive neuroscience* **20**, 95-107 (2008).
19. W. Li, A. Keil, Sensing fear: fast and precise threat evaluation in human sensory cortex. *Trends in cognitive sciences* **27**, 341-352 (2023).
20. Z. Li, A. Yan, K. Guo, W. Li, Fear-related signals in the primary visual cortex. *Current Biology* **29**, 4078-4083. e4072 (2019).
