# Supplementary material for: Trait-like visual cortical hyperactivity in trait anxiety
Source: Nat Commun. 2025 Dec 20;17:794. doi: 10.1038/s41467-025-67480-3 (PMC12824385; doi:10.1038/s41467-025-67480-3)
Supplement: Supplementary file 2 — Reporting Summary [file 41467_2025_67480_MOESM2_ESM.pdf]

nature portfolio

Corresponding author(s): Wen Li

Last updated by author(s): Nov. 5, 2025

Reporting Summary

Nature Portfolio wishes to improve the reproducibility of the work that we publish. This form provides structure for consistency and transparency in reporting. For further information on Nature Portfolio policies, see our Editorial Policies and the Editorial Policy Checklist.

Statistics

For all statistical analyses, confirm that the following items are present in the figure legend, table legend, main text, or Methods section.

n/a	Confirmed	
☐	☒	The exact sample size (*n*) for each experimental group/condition, given as a discrete number and unit of measurement
☐	☒	A statement on whether measurements were taken from distinct samples or whether the same sample was measured repeatedly
☐	☒	The statistical test(s) used AND whether they are one- or two-sided<br>*Only common tests should be described solely by name; describe more complex techniques in the Methods section.*
☒	☐	A description of all covariates tested
☐	☒	A description of any assumptions or corrections, such as tests of normality and adjustment for multiple comparisons
☐	☒	A full description of the statistical parameters including central tendency (e.g. means) or other basic estimates (e.g. regression coefficient) AND variation (e.g. standard deviation) or associated estimates of uncertainty (e.g. confidence intervals)
☐	☒	For null hypothesis testing, the test statistic (e.g. *F*, *t*, *r*) with confidence intervals, effect sizes, degrees of freedom and *P* value noted<br>*Give P values as exact values whenever suitable.*
☐	☒	For Bayesian analysis, information on the choice of priors and Markov chain Monte Carlo settings
☐	☒	For hierarchical and complex designs, identification of the appropriate level for tests and full reporting of outcomes
☐	☒	Estimates of effect sizes (e.g. Cohen's *d*, Pearson's *r*), indicating how they were calculated

Our web collection on statistics for biologists contains articles on many of the points above.

Software and code

Policy information about availability of computer code

Data collection	A section of "Data availability" is included, indicating data are deposited on GitHub (https://github.com/LiLabFSU/).
Data analysis	A section of "Code availability" is included, indicating code are deposited on GitHub (https://github.com/LiLabFSU/).

For manuscripts utilizing custom algorithms or software that are central to the research but not yet described in published literature, software must be made available to editors and reviewers. We strongly encourage code deposition in a community repository (e.g. GitHub). See the Nature Portfolio guidelines for submitting code & software for further information.

Data

Policy information about availability of data

All manuscripts must include a data availability statement. This statement should provide the following information, where applicable:

- Accession codes, unique identifiers, or web links for publicly available datasets
- A description of any restrictions on data availability
- For clinical datasets or third party data, please ensure that the statement adheres to our policy

Data are now provided with this paper as deposited on Github

Research involving human participants, their data, or biological material

Policy information about studies with human participants or human data. See also policy information about sex, gender (identity/presentation), and sexual orientation and race, ethnicity and racism.

Reporting on sex and gender	Both sexes were included in the study; There was no specific a priori hypothesis regarding the effect of sex on the association between sensory cortical hyperactivity and trait anxiety. Therefore, sex was not considered as a key variable in the study design. As per a reviewer's suggestion, we performed exploratory analysis of sex effects in the resubmission. Sex was determined based on self-report.
Reporting on race, ethnicity, or other socially relevant groupings	Socially constructed or relevant categorization variables were not included in the study.
Population characteristics	No covariates were included in the study.
Recruitment	Participants were recruited from communities in Madison, WI, and Tallahassee, FL, through standard channels, including flyers, advertisements, and classroom presentations. There is no indication of self-selection bias or other biases.
Ethics oversight	The IRB of University of Wisconsin-Madison and the IRB of Florida State University

Note that full information on the approval of the study protocol must also be provided in the manuscript.

Field-specific reporting

Please select the one below that is the best fit for your research. If you are not sure, read the appropriate sections before making your selection.

☒ Life sciences ☐ Behavioural & social sciences ☐ Ecological, evolutionary & environmental sciences

For a reference copy of the document with all sections, see nature.com/documents/nr-reporting-summary-flat.pdf

Life sciences study design

All studies must disclose on these points even when the disclosure is negative.

Sample size	The sample sizes for the four experiments ranged 46-52. For each experiment, the sample size was determined based on a power analysis for a medium effect size typically observed for trait anxiety (r = .35), which requires a sample size of 46 to achieve a power of .80.
Data exclusions	A small number of participants were excluded due to excessive eye movements, severe EEG artifact, and technical problems.
Replication	The four experiments included built-in replications of key effects to assess reproducibility. Indeed, they yielded consistent results, providing strong support for reproducibility.
Randomization	There was no experimental group assignment. No covariates were included.
Blinding	All participants underwent the same procedures, with no condition allocation across participants. Blinding was deemed not necessary.

Reporting for specific materials, systems and methods

We require information from authors about some types of materials, experimental systems and methods used in many studies. Here, indicate whether each material, system or method listed is relevant to your study. If you are not sure if a list item applies to your research, read the appropriate section before selecting a response.

Materials & experimental systems

n/a	Involved in the study	
☒	☐	Antibodies
☒	☐	Eukaryotic cell lines
☒	☐	Palaeontology and archaeology
☒	☐	Animals and other organisms
☒	☐	Clinical data
☒	☐	Dual use research of concern
☒	☐	Plants

Methods

n/a	Involved in the study	
☒	☐	ChIP-seq
☒	☐	Flow cytometry
☒	☐	MRI-based neuroimaging

Plants

Seed stocks	n/a
Novel plant genotypes	n/a
Authentication	n/a
