# Supplementary material for: Trait-like visual cortical hyperactivity in trait anxiety
Source: Nat Commun. 2025 Dec 20;17:794. doi: 10.1038/s41467-025-67480-3 (PMC12824385; doi:10.1038/s41467-025-67480-3)
Supplement: Supplementary file 4 — Source Data [file 41467_2025_67480_MOESM4_ESM.zip › Source_data_intro.docx]

**Figure 1:**

Sub: subject ID

Sex: 1 = male, 0 = female

BIS: trait anxiety score

T1/2/3 P1: P1 amplitude at time 1, 2, 3 at Oz site when presenting M-selective Gabor patches

T1/2/3 C1N1: C1-N1 amplitude at time 1, 2, 3 at Oz site when presenting P-selective Gabor patches

**Figure 2:**

Sub: subject ID

Sex: 1 = male, 0 = female

Bistotal: trait anxiety score

ML_P1_P8: P1 amplitude at P8 site when M-selective Gabor patches is on the left in experiment 2

MR_P1_P7: P1 amplitude at P7 site when M-selective Gabor patches is on the right in experiment 2

PC_C2_Oz: C1 amplitude at Oz site when P-selective Gabor patches is in the middle in experiment 2

LSF_P1_Oz: P1 amplitude at Oz site when presenting M-selective Gabor patches in experiment 3

HSF_C1_Oz: C1 amplitude at Oz site when presenting P-selective Gabor patches in experiment 3

EO_ap_exp: aperiodic exponent during resting-state at electrodes around Cz

Note: The source data for source-localization cannot be given directly since it is conducted in eLORETA software, but can be recreated with the EEG data provided and also eLORETA software.

**Figure 3:**

Sub: subject ID

Sex: 1 = male, 0 = female

BIS: trait anxiety score

BIS_zscore: BIS score that is z-transformed

LSF_P1_Oz: P1 amplitude at Oz site when presenting M-selective stimuli in experiment 4

HSF_P1_Oz: P1 amplitude at Oz site when presenting P-selective stimuli in experiment 4

**Table S1:**

Sub: subject ID

BIS: trait anxiety score

BIS_zscore: BIS score that is z-transformed

CSp_P1_T1/2/3: P1 amplitude at time 1, 2, 3 at Oz site when presenting CS+ M-selective Gabor patches

CSm_P1_T1/2/3: P1 amplitude at time 1, 2, 3 at Oz site when presenting CS- M-selective Gabor patches

CSp_C1N1_T1/2/3: C1-N1 amplitude at time 1, 2, 3 at Oz site when presenting CS+ P-selective Gabor patches

CSm_ C1N1_T1/2/3: C1-N1 amplitude at time 1, 2, 3 at Oz site when presenting CS- P-selective Gabor patches

**Figure S1:**

Sub: subject ID

Sex: 1 = male, 0 = female

BIS: trait anxiety score

T1/2/3 P1: P1 amplitude at time 1, 2, 3 at Oz site when presenting M-selective Gabor patches

T1/2/3 C1N1: C1-N1 amplitude at time 1, 2, 3 at Oz site when presenting P-selective Gabor patches

Notes: this dataset is the same as Figure 1 dataset

**Figure S2-4:**

The source data for source-localization cannot be given directly since it is conducted in eLORETA software, but can be recreated with the EEG data provided and also eLORETA software.
